# Supplementary material for: Controllable modulation of precursor reactivity using chemical additives for systematic synthesis of high-quality quantum dots
Source: Nat Commun. 2020 Nov 12;11:5748. doi: 10.1038/s41467-020-19573-4 (PMC7665041; doi:10.1038/s41467-020-19573-4)
Supplement: Supplementary file 1 — Supplementary Information [file 41467_2020_19573_MOESM1_ESM.pdf]

Supporting information

Controllable Modulation of Precursor Reactivity Using Chemical Additives for Systematic Synthesis of High-Quality Quantum Dots

Authors: Joonhyuck Park[1], Arun Jayaraman[1], Alex W. Schrader[1], Gyu Weon Hwang[2], Hee-Sun Han[1,3,4]

Dr. J. Park, A. Jayaraman, A. W. Schrader, and Prof. H-.S. Han[1]
[1]Department of Chemistry, University of Illinois at Urbana-Champaign, 600 South Mathews Avenue, Urbana, Illinois 61801, United States
Dr. G. W. Hwang[2]
[2]Center for Electronic Materials, Korea Institute of Science and Technology, Seoul, 02792, Korea
Prof. H-.S. Han[3,4]
[3]The Carl R. Woese Institute for Genomic Biology (IGB), University of Illinois at Urbana-Champaign, 1206 W Gregory Dr, Urbana, Illinois 61801, United States
[4]Center for Biophysics and Quantitative Biology, University of Illinois at Urbana-Champaign, 600 South Mathews Avenue, Urbana, Illinois 61801, United States

Materials and methods:

Materials: n-tetradecylphosphonic acid (TDPA, 97%), oleylamine (OAm, 70%), 1-octadecene (ODE, 90%), trioctylphosphine oxide (TOPO, 99%), trioctylphosphine (TOP, 97%), oleic acid (OA, 90%), hexadecylamine (HDA, 90%), diphenylphosphine (DPP, 98%), 4-(dimethylamino)pyridine (DMAP, 99%), picoline (99%), 3-chloropyridine (99%), 4-cyanopyridine (98%), 1,4-diazabicyclo[2.2.2]octane (DABCO, 99%), 1-methylpyrolidine (98%), 1-phenylimidazole (97%), hydrogen sulfide solution (0.8 M in tetrahydrofuran), octanethiol (OT, 98.5%), diphenyl ether (99%), lead(II) oxide (99.999%), trifluoroacetic anhydride (99%), trifluoroacetic acid (99%), aniline (99%), phenyl isothiocyantate (98%), indium(III) acetate (99.99%), copper(I) iodide (99.999%), dodecanethiol (DDT, >98%), selenium dioxide (SeO_2, 99.9+%), myristic acid (99%), and 9-borabicyclo[3.3.1]nonane dimer were purchased from Sigma Aldrich. 4-(trifluoromethyl)pyridine (99%) was purchased from Oakwood Chemical. Selenium shot, octadecylphosphonic acid (ODPA, 97%), cadmium oxide (CdO, 99.999%), and hexyl isothiocyanate (97%) were purchased from Alfa Aesar. Tributylphosphine (TBP) was purchased from Strem Chemicals. Bis(trimethylsilyl)sulfide ($(TMS)_2S$) and dodecylamine (98%) were purchased from Acros Organics. All reagents were used as received unless noted. Toluene was prepared by a solvent purification system (SPS). Other solvents were of reagent grade or higher and were used without further purification. 1.5 M trioctylphosphine selenide (TOP-Se) was prepared by dissolving 0.15 mmol of selenium shot in 100 mL of TOP under inert atmosphere and stirring vigorously overnight, forming a 1.5 M TOPSe solution. BBN-SH, $Pb(oleate)_2$, 1,3-diphenylimidazolidine-2-selenone (Se-Im(Ph_2)), N-*n*-hexyl-N'-dodecylthiourea, and N,N'-diphenylthiourea were synthesized using the previously reported method.[1,2] All air sensitive materials were handled in a glove box under dry nitrogen atmosphere with oxygen levels < 0.1 ppm.

TEM measurement: Samples for TEM were prepared by dropping on a TEM grid and drying highly concentrated pure QDs in the mixture of chloroform and toluene. TEM images and selected area electron diffraction (SAED) patterns were acquired using JEOL 2010 LaB6 operating at 200 kV in the Frederick Seitz Materials Research Laboratory Central Research Facilities at UIUC.

XRD measurement and pattern simulation: Samples for XRD were prepared by dropping and drying highly concentrated pure QDs in hexane on quartz holder. XRD patterns were acquired using a Rigaku MiniFlex 600 in the George L. Clark X-Ray Facility and 3M Materials Laboratory at UIUC. The instrument was equipped with a Cu Kα line (λ= 1.5406 Å) X-ray source operating at 40 kV, 15 mA and scattering signals were collected in θ–2θ scan mode in 3~100° 2θ range with 0.4° min^{-1} scanning speed. Powder XRD pattern for nanoparticles and bulk were simulated by DIFFaX v1.812 in different degrees of stacking fault.

$Pb(oleate)_2$ synthesis: $Pb(oleate)_2$ was synthesized using a previously reported method.[1] 22.4 mmol (5.00 g) of lead(II) oxide and 10 mL of acetonitrile are mixed in a 100 mL round bottom flask. The suspension is stirred while being cooled in an ice bath for 5 minutes. 4.48 mmol (0.35 mL, 0.2 eq to lead(II) oxide) of trifluoroacetic acid and 22.4 mmol (3.1 mL, 1 eq to lead(II) oxide) of trifluoroacetic anhydride were added to the round bottom flask. The yellow lead oxide was dissolved, resulting in a clear and colorless lead trifluoroacetate solution that is allowed to warm to room temperature for 20 minutes. To another 250 mL round bottom flask, 45.2 mmol (12.78 g, 2.02 eq to lead(II) oxide) of oleic acid, 90 mL of isopropanol, and 50.6 mmol of triethylamine (5.12 g, 2.26 eq to lead(II) oxide) are added. The lead trifluoroacetate solution is then added to the oleic acid solution slowly with stirring, resulting in the formation of a white precipitate. The mixture is heated to dissolve the precipitate and results in a clear and colorless solution. The clear solution allowed to slowly cool to room temperature for 1 hour, followed by further cooling in a -20 °C freezer overnight. The resulting white powder is isolated by suction filtration using a glass fritted funnel. The filtrate thoroughly washed with 300 mL of methanol three times. The white powder is dried under vacuum for 6 hours. The resulted $Pb(oleate)_2$ powder is stored in a nitrogen-filled glovebox. The yield of $Pb(oleate)_2$ powder was 16.4 g (95%).

9-mercapto-9-borabicyclo[3.3.1]nonane (BBN-SH) synthesis: 9-mercapto-9-borabicyclo[3.3.1]nonane (BBN-SH) was synthesized using a previously reported method.[3] Under the inert gas environment, 10 mmol (2.44 g) of 9-borabicyclo[3.3.1]nonane (9-BBN) dimer was placed in a flask. 50 mL of toluene was freshly purified by SPS and added in the flask. The flask with the mixture of 9-BBN and toluene was sealed and connected under a nitrogen flow from Schlenk line. 25 mL of H_2S solution (0.8 M in tetrahydrofuran) was added slowly to the flask and heated up to 80°C. After the solution become clear, the solution was refluxed overnight. The residual solvent was removed by rotary evaporation. Pure BBN-SH (2.72 g, 89%) was isolated by vacuum sublimation. ^1H NMR (ppm, $CDCl_3$, 400 MHz): δ = 3.71 (s, H, -SH), 2.20-1.13 (m, 14H, $-BC_8H_{14}$); ^{11}B NMR (ppm, $CDCl_3$, 400 MHz): δ =61.2 (s)

Synthesis of N,N'-diphenylthiourea: N,N'-diphenylthiourea was synthesized using a previously reported method.[1] Briefly, 30 mmol (2.79 g) of aniline in 10 mL of toluene and phenyl isothiocyanate (4.06 g, 30 mmol) in another 10 mL of toluene were mixed, resulting in a white powder. Toluene was removed by a rotary evaporator. Residual toluene was removed under vacuum for 2 hours. Yield: 6.71 g (98%). ^1H NMR (ppm, C_6D_6, 400 MHz): δ = 7.01 (m, 10H, $-C_6H_5$), 7.59 (s, 2H, NH).

Synthesis of N-*n*-hexyl-N'-dodecylthiourea: N-*n*-hexyl-N'-dodecylthiourea was synthesized using a previously reported method.[1] Briefly, 30 mmol (5.56 g) of dodecylamine in 10 mL of toluene and hexyl

isothiocyanate (4.30 g, 30 mmol) in another 10 mL of toluene were mixed, resulting in a white powder. Toluene was removed by a rotary evaporator. Residual toluene was removed under vacuum for 2 hours. Yield: 9.39 g (95%). ^{1}H NMR (C_6D_6, 400 MHz): δ = 0.86 (t, 3H, $-CH_3$), 0.91 (t, 3H, $-CH_3$), 1.11-1.40 (m, 28H, $(CH_2)_{10}$ and $(CH_2)_4$), 3.21 (b, 4H, $-CH_2$), 5.42 (b, 2H, NH)

Quantum yield (QY) measurement: QYs of CdSe/CdS QDs were measured relative to rhodamine 560, rhodamine 640, and cresyl violet 670. Fluorescence spectra of QD and dye were taken under identical spectrometer conditions in triplicate and averaged. The optical density was kept below 0.1 between 300 and 700 nm, and the integrated intensities of the emission spectra, corrected for differences in index of refraction and concentration, were used to calculate the quantum yields using the expression $QY_{QD} = QY_{Dye} \times (\text{absorbance}_{dye}/\text{absorbance}_{QD}) \times (\text{peak area}_{QD}/\text{peak area}_{Dye}) \times (n_{QD}\ \text{solvent})^2/(n_{Dye}\ \text{solvent})^2$ (n: refractive index of each solvent).

Single QD blinking measurement: All the single-particle measurements were performed under an ambient condition. Samples were prepared by spin-coating highly diluted QD solutions in hexane on glass coverslips. Single particle traces were acquired using the Fluorescence Fluctuation Spectroscopy (FFS) module on an Alba system (ISS Inc., Urbana, IL). An avalanche photodiode (APD) detector was used acquire single QD photoluminescence (PL) traces at 200,000 data points per second. QDs were observed using a 100x, 1.46 NA oil objective (420792-9800-000, Zeiss), excited using a 488 nm CW laser with a power of 80 nW, and observed through a 630±75 nm band pass filter (Zeiss). Data was acquired using VistaVison (ISS Inc.), and processed using Origin 2019 (Origin Lab).

Wurtzite CdSe core synthesis: CdSe cores with 455 nm first absorption peak (small size) were synthesized using a previously reported method.[4] To summarize, 0.44 mmol (60 mg) of CdO, 0.88 mmol (0.2475g) of TDPA, 5 mL of ODE, and 2 g of HDA were placed in 25 mL round bottom flask. The solution was degassed for 1 hr at 100 °C and heated to 310 °C under nitrogen until the CdO dissolved and formed a clear homogenous solution. 4.5 mL of TOP, 0.3 mmol (55.9 mg) of DPP, and 2 mL of 1.5 M TOPSe solution was rapidly injected. The cores were then grown further at 270 °C to produce cores with 455 nm for the first absorption feature.

CdSe cores with 535 nm first absorption peak (medium size) were synthesized using a previously reported method.[4] To summarize, 0.44 mmol (60 mg) of CdO, 0.88 mmol (0.2475g) of TDPA, 9.56 mmol (3.70 g) of TOPO were placed in 25 mL round bottom flask. The solution was degassed for 1 hr at 120 °C and heated to 320 °C under nitrogen until the CdO dissolved and formed a clear homogenous solution. 1 mL of TOP was injected and the solution was reheated to 330°C under nitrogen. 1.5 mL of 1.5 M TOP-Se solution was rapidly injected. The cores were then grown further at 280°C to produce cores with 535 nm for the first absorption feature.

CdSe cores with 580 nm first absorption peak (big size) were synthesized using a previously reported method.[4] To summarize, 0.44 mmol (60 mg) of CdO, 0.88 mmol (0.2475g) of TDPA, 9.56 mmol (3.70 g) of TOPO were placed in 25 mL round bottom flask. The solution was degassed for 1 hr at 120 °C and heated to 320 °C under nitrogen until the CdO dissolved and formed a clear homogenous solution. 1 mL of TOP was injected and the solution was reheated to 350°C under nitrogen. 1.5 mL of 1.5 M TOP-Se solution was rapidly injected. The cores were then grown further at 280°C to produce cores with the 580 nm for the first absorption feature.

Zinc-blende CdSe core synthesis: Zinc-blende CdSe cores with 580 nm first absorption peak were synthesized using a previously reported method with a minor modification.[5] In general, 0.1 mmol of selenium dioxide powder and 0.1 mmol of cadmium myristate were added to a 25 mL round bottom flask with 5 mL of ODE. The mixture was degassed for 10 min under vacuum at room temperature. Under argon flow, the solution was stirred and heated to 270 ºC. After 2 minutes of growth, 0.2 mL of oleic acid was added dropwise into the reaction solution to stabilize the growth of the nanocrystals. The reaction was monitored by UV-Vis spectroscopy and was stopped by removing the heat when the QDs reached a desired size.

Evaluation of the melting temperature of CdSe QD cores: 50 nmol of CdSe cores (small cores: 1.8 nm (in diameter), medium: 3.6 nm, big: 4.6 nm) were isolated by repeated precipitations from hexane with acetone. The CdSe QD cores were redispersed in a minimal amount of hexane and loaded in a solvent mixture of 3 mL of OAm and 3 mL of ODE. The reaction solution was degassed under vacuum at 100 °C for 1 hr. To determine the melting temperature, the reaction temperature for the CdSe cores and shell precursors was slowly increased by 10 ºC over 20 min and maintained for another 20 min.

Identification of the optimal activation temperature for the shell growth: CdS shell growth on medium size CdSe core (535 nm absorption peak) using BBN-SH. 50 nmol of CdSe cores were isolated by repeated precipitations from hexane with acetone. The CdSe QD cores were redispersed in a minimal amount of hexane and loaded in a solvent mixture of 3 mL of OAm and 3 mL of ODE. The reaction solution was degassed under vacuum at 100 °C for 1 hr. The temperature was increased to 170 °C. The amount of each Cd or S precursor was calculated for growing 7 ML of CdS shell on the CdSe QD cores. The Cd precursor (0.28 mmol Cd-oleate in 6 mL of ODE) and the S precursor (0.28 mmol of BBN-SH in 0.28 mmol of 3-chloropyridine, 1 mL of OAm, 0.95 mL of TOP, and 4 mL of ODE) were slowly injected (3 mL/hr) to the CdSe core solution simultaneously. The reaction temperature was maintained for 10 min and increased by 5ºC every 5 minutes up to 200ºC. The reaction temperature was kept at 200ºC for another 80 min. After the 2 hr precursor injection, the reaction vessel was cooled down to r.t. For the picoline case, all parameters were the same as above. For the 4-cyanopyridine case, the reaction temperature was kept at 190ºC and increased 5ºC by every 15 minutes up to 200ºC. Other parameters were the same as above.

CdS shell growth kinetics study: 50 nmol of CdSe cores were isolated by repeated precipitations from hexane with acetone. The CdSe QD cores were redispersed in a minimal amount of hexane and loaded in a solvent mixture of 3 mL of OAm and 3 mL of ODE. The reaction solution was degassed under vacuum at 100 °C for 1 hr and dropped to 60 °C. The amount of each Cd or S precursor was calculated for growing 7 ML of CdS shell on the CdSe QD cores. The Cd precursor (0.28 mmol Cd-oleate in ODE) and the S precursor (0.28 mmol of BBN-SH in 1 mL of OAm, and 1 mL of TOP) were injected to the CdSe core solution sequentially. The reaction temperature for the mixture of CdSe cores and shell precursors was brought to 170ºC and maintained for 20 min. 0.28 mmol of each chemical initiator (DMAP, picoline, 3-chloropyridine) in 0.5 mL OAm was added in the mixture. For the control sample, no chemical initiator was added. To determine the CdS shell growth kinetics by different types of activators, the reaction temperature was kept at 170ºC and the emission spectrum (the peak wavelength and FWHM of the photoluminescence spectrum) was recorded every 10 min.

CdS shell growth on small size CdSe core (455 nm absorption peak) using BBN-SH: 50 nmol of CdSe cores were isolated by repeated precipitations from hexane with acetone. The CdSe QD cores were redispersed in a minimal amount of hexane and loaded in a solvent mixture of 3 mL of OAm and 3 mL of

ODE. The reaction solution was degassed under vacuum at 100 °C for 1 hr. The amount of each Cd or S precursor was calculated for growing 7 ML of CdS shell on the CdSe QD cores. The Cd precursor (0.15 mmol Cd-oleate in 6 mL of ODE) and the S precursor (0.15 mmol of BBN-SH in 0.15 mmol of DMAP, 1 mL of OAm, 1 mL of TOP, and 4 mL of ODE) were slowly injected (3 mL/hr) to the CdSe core solution simultaneously. The reaction temperature was maintained for 10 min, then increased by 5°C every 5 minutes up to 130°C. The reaction temperature was kept at 130°C for another 80 min. After the 2 hr precursor injection, the reaction vessel was cooled down to r.t. To assess how the reaction temperature impacts on the quality of resulted core/shell QDs, [BBN-SH:picoline] pair used at 140-170°C or [BBN-SH:3-ClPy] pair used at 170-200°C while keeping all other procedures the same as above.

CdS shell growth on medium size CdSe core (535 nm absorption peak) using BBN-SH: 50 nmol of CdSe cores were isolated by repeated precipitations from hexane with acetone. The CdSe QD cores were redispersed in a minimal amount of hexane and loaded in a solvent mixture of 3 mL of OAm and 3 mL of ODE. The reaction solution was degassed under vacuum at 100 °C for 1 hr. The temperature was increased to 170 °C. The amount of each Cd or S precursor was calculated for growing 7 ML of CdS shell on the CdSe QD cores. The Cd precursor (0.28 mmol Cd-oleate in 6 mL of ODE) and the S precursor (0.28 mmol of BBN-SH in 0.28 mmol of 3-chloropyridine, 1 mL of OAm, 0.95 mL of TOP, and 4 mL of ODE) were slowly injected (3 mL/hr) to the CdSe core solution simultaneously. The reaction temperature was maintained for 10 min, then increased by 5°C every 5 minutes up to 200°C. The reaction temperature was kept at 200°C for another 80 min. After the 2 hr precursor injection, the reaction vessel was cooled down to r.t.

CdS shell growth on big size CdSe core (580 nm absorption peak) using BBN-SH: 50 nmol of CdSe cores were isolated by repeated precipitations from hexane with acetone. The CdSe QD cores were redispersed in a minimal amount of hexane and loaded in a solvent mixture of 3 mL of OAm and 3 mL of ODE. The reaction solution was degassed under vacuum at 100 °C for 1 hr. The temperature was increased to 200 °C. The amount of each Cd or S precursor was calculated for growing 7 ML of CdS shell on the CdSe QD cores. The Cd precursor (0.41 mmol Cd-oleate in 6 mL of ODE) and the S precursor (0.41 mmol of BBN-SH in 0.41 mmol of 4-(trifluoromethyl)pyridine, 1 mL of OAm, 0.9 mL of TOP, and 4 mL of ODE) were slowly injected (3 mL/hr) to the CdSe core solution simultaneously. The reaction temperature was maintained for 10 min, then increased by 5°C every 5 minutes up to 230°C. The reaction temperature was kept at 230°C for another 80 min. After the 2 hr precursor injection, the reaction vessel was cooled down to r.t. To assess how the precursor reactivity impacts on the quality of resulted core/shell QDs, [BBN-SH:DMAP] pair used for 2 hr precursor injection or [BBN-SH:DMAP] pair used for 24 hr precursor injection while keeping all other procedures the same as above.

Asymmetric QD growth on big size CdSe core (580 nm absorption peak) using BBN-SH: 50 nmol of CdSe cores were isolated by repeated precipitations from hexane with acetone. Either zinc-blende or wurtzite CdSe QD cores were redispersed in a minimal amount of hexane and loaded in a mixture of 0.1 mmol of ODPA, 3 mL of OAm, and 3 mL of ODE. The reaction solution was degassed under vacuum at 100 °C for 1 hr. The temperature was increased to 140 °C. The amount of each Cd or S precursor was calculated for growing 7 ML of CdS shell on the CdSe QD cores. The Cd precursor (0.41 mmol Cd-oleate in 6 mL of ODE) and the S precursor (0.41 mmol of BBN-SH in 0.41 mmol of DMAP, 1 mL of OAm, 0.9 mL of TOP, and 4 mL of ODE) were slowly injected (3 mL/hr) to the CdSe core solution simultaneously. The reaction temperature was maintained for 2 hr. To assess how the ligand composition impacts on the

faceted growth of resulted core/shell QDs, ODPA was removed from the reaction vessel while keeping all other procedures the same as above.

CdS shell growth on small size CdSe core (455 nm absorption peak) using $(TMS)_2S$: 50 nmol of CdSe cores were isolated by repeated precipitations from hexane with acetone. The CdSe QD cores were redispersed in a minimal amount of hexane and loaded in a solvent mixture of 3 mL of OAm and 3 mL of ODE. The reaction solution was degassed under vacuum at 100 °C for 1 hr. The amount of each Cd or S precursor was calculated for growing 7 ML of CdS shell on the CdSe QD cores. The Cd precursor (0.15 mmol Cd-oleate, 0.3 mmol of OAm, and 5.9 mL of TOP) and the S precursor (0.15 mmol of $(TMS)_2S$ in 6 mL of TOP) were slowly injected (3 mL/hr) to the CdSe core solution simultaneously. The reaction temperature was maintained for 10 min, then increased by 5°C every 5 minutes up to 130°C. The reaction temperature was kept at 130°C for another 80 min. After the 2 hr precursor injection, the reaction vessel was cooled down to r.t.

CdS shell growth on small size CdSe core (455 nm absorption peak) using octanethiol: 50 nmol of CdSe cores were isolated by repeated precipitations from hexane with acetone. The CdSe QD cores were redispersed in a minimal amount of hexane and loaded in a solvent mixture of 3 mL of OAm and 3 mL of ODE. The reaction solution was degassed under vacuum at 100 °C for 1 hr. The temperature was increased to 240 °C. The amount of each Cd or S precursor was calculated for growing 7 ML of CdS shell on the CdSe QD cores. The Cd precursor (0.15 mmol Cd-oleate in 6 mL of ODE) and the S precursor (0.15 mmol of octanethiol in 6 mL of ODE) were slowly injected (3 mL/hr) to the CdSe core solution simultaneously. The reaction temperature was increased by 7°C every in 1 minute up to 310°C. The reaction temperature was kept at 310°C for another 110 min. After the 2 hr precursor injection, the reaction vessel was cooled down to r.t.

CdS shell growth on big size CdSe core (580 nm absorption peak) using $(TMS)_2S$: 50 nmol of CdSe cores isolated by repeated precipitations from hexane with acetone. The CdSe QD cores were redispersed in a minimal amount of hexane and loaded in a solvent mixture of 3 mL of OAm and 3 mL of ODE. The reaction solution was degassed under vacuum at 100 °C for 1 hr. The temperature was increased to 130 °C. The amount of each Cd or S precursor was calculated for growing 7 ML of CdS shell on the CdSe QD cores. The Cd precursor (0.41 mmol Cd-oleate, 0.82 mmol of OAm in 5.7 mL of TOP) and the S precursor (0.41 mmol of $(TMS)_2S$ in 6 mL of TOP) were slowly injected (3 mL/hr) to the CdSe core solution simultaneously. The reaction temperature was maintained for 10 min, then increased by 5°C every 5 minutes up to 130°C. The reaction temperature was kept at 130°C for another 80 min. After the 2 hr precursor injection, the reaction vessel was cooled down to r.t.

CdS shell growth on big size CdSe core (580 nm absorption peak) using octanethiol: 50 nmol of CdSe cores isolated by repeated precipitations from hexane with acetone. The CdSe QD cores were redispersed in a minimal amount of hexane and loaded in a solvent mixture of 3 mL of OAm and 3 mL of ODE. The reaction solution was degassed under vacuum at 100 °C for 1 hr. The temperature was increased to 240 °C. The amount of each Cd or S precursor was calculated for growing 7 ML of CdS shell on the CdSe QD cores. The Cd precursor (0.41 mmol Cd-oleate in 6 mL of ODE) and the S precursor (0.41 mmol of octanethiol in 6 mL of ODE) were slowly injected (3 mL/hr) to the CdSe core solution simultaneously. The reaction temperature was increased by 7°C every in 1 minute up to 310°C. The reaction temperature was kept at 310°C for another 110 min. After the 2 hr precursor injection, the reaction vessel was cooled down to r.t.

PbS QDs synthesis and PbS QDs size tuning with BBN-SH: PbS QD cores were synthesized by using a previously reported method with modification.[1] Briefly, 0.11 mmol (88 mg) of Pb(oleate)$_2$, 0.22 mmol (65 mg) of OA, and 2 mL of ODE were degassed under 100°C for 1 hr. 0.76 mmol (17 mg) of N,N'-diphenylthiourea in 300 μL of diphenyl ether was injected at 95°C. Nucleation is started within 60 seconds. After 2 minutes for growing further PbS QD cores, the heating mantle was removed and the reaction vessel was cooled down quickly to room temperature. Crude PbS QDs were purified by adding toluene and ethyl acetate (toluene:ethyl acetate = 1:3 (v/v)) 2 times. The concentration of PbS QD was determined by the absorbance at 400 nm, where the extinction is proportional to the concentration of PbS QDs for different sizes of PbS QDs.[6] 50 nmol of PbS QD cores were transferred in 3 mL of OAm and 3 mL of ODE. The reaction solution was degassed under vacuum at 100 °C for 1 hr and the temperature was kept at 100°C. The Pb precursor (0.21 mmol Pb(oleate)$_2$ in 6 mL of ODE) and the S precursor (0.21 mmol of BBN-SH in 0.21 mmol of DMAP, 1 mL of OAm, 0.9 mL of TOP, 4 mL of ODE) were slowly injected (3 mL/hr) to the PbS QD solution simultaneously. The reaction temperature was maintained for 10 min and increased by 10°C every 10 minutes up to 130°C. After the precursor injection for 2 hrs, the reaction vessel was cooled down to r.t.

$CuInS_2$ QD synthesis and tuning the size of $CuInS_2$ QDs with BBN-SH: $CuInS_2$ QD cores were synthesized by using a previously reported method with modification.[7] Briefly, 0.5 mmol (95 mg) of copper(I) iodide and 0.5 mmol (146 mg) of indium (III) acetate, and 3 mL of dodecanethiol (DDT) were employed as precursors and solvent. 1.5 g of OA and 2 mL of ODE were also added. Under vacuum, the reaction was heated for 30 min at 100 °C. Then the temperature was raised to 220 °C. After 10 minutes of growing further $CuInS_2$ QDs cores, the heating mantle was removed and the reaction vessel was cooled down quickly to room temperature. Crude $CuInS_2$ QDs were purified by adding acetone and methanol 2 times. 50 nmol of $CuInS_2$ QD cores were transferred in 3 mL of OAm and 3 mL of ODE. The reaction solution was degassed under vacuum at 100 °C for 1 hr and the temperature was kept at 170°C. The Cu and In precursor (0.11 mmol Cu(oleate), 0.11 mmol In(oleate)$_3$ in 6 mL of ODE) and the S precursor (0.11 mmol of BBN-SH in 0.11 mmol of 4-ClPy, 1 mL of OAm, 0.9 mL of TOP, 4 mL of ODE) were slowly injected (3 mL/hr) to the $CuInS_2$ QD solution simultaneously. The reaction temperature was kept for 10 min and increased by 10°C every 10 minutes up to 200°C. After the precursor injection for 2 hr, the reaction vessel was cooled down to r.t.

Supplementary results:

1. Asymmetric QD growth for Figure S8: Even though the asymmetric QD growth was not the main scope of our paper, we did further experiments on the facet dependent QD growth in the presence of surface-selective binding ligands and low reaction temperature condition. We expected to achieve the anisotropic growth of shell under low temperature condition, where the difference between the surface free energy of each facet in QD can be high enough to induce the facet dependent growth. Under low temperature condition, however, our precursor by themselves did not show the distinctive facet development on QD surface during the shell growth. We attribute this result to small differences in the surface energy of different facets. To maximize the difference in surface energy, we added octadecylphosphonic acid, which is known to strongly bind on certain facet (($11\bar{2}0$) facet of CdSe) and lower the surface energy.[8,9] As expected, we have synthesized the elongated CdSe/CdS nanorods from our wurzite CdSe QD cores and the CdSe/CdS tetrapods from our zinc blende CdSe QD core in the presence of phosphonic acids.

2. Precursor reactivity of thiourea precursors for Figure S11: We compared the precursor reactivity between thiourea precursors and BBN-SH precursor by measuring the temperature that initiate the CdS shell on CdSe QD core. To find their temperature range for initiating the CdS shell growth, we choose two different thiourea precursors with a wide range of conversion rate (k) (N-*n*-hexyl-N'-dodecylthiourea (k: 1) and N,N'-diphenylthiourea (k: 1100)). A red shift of the PL peak of CdSe/CdS core/shell QDs indicate the CdS shell growth. We found that a relatively low temperature (60-110°C) for CdS shell growth results inefficient CdS shell growth and generates satellite particles easily. This result indicates a high-quality shell growth using thiourea precursors is not ideal for CdS shell growth in a wide range of the reaction temperature with various size of CdSe QD core. In comparison, our BBN-SH precursor can initiate the CdS shell growth in a wide temperature range (100-230°C).

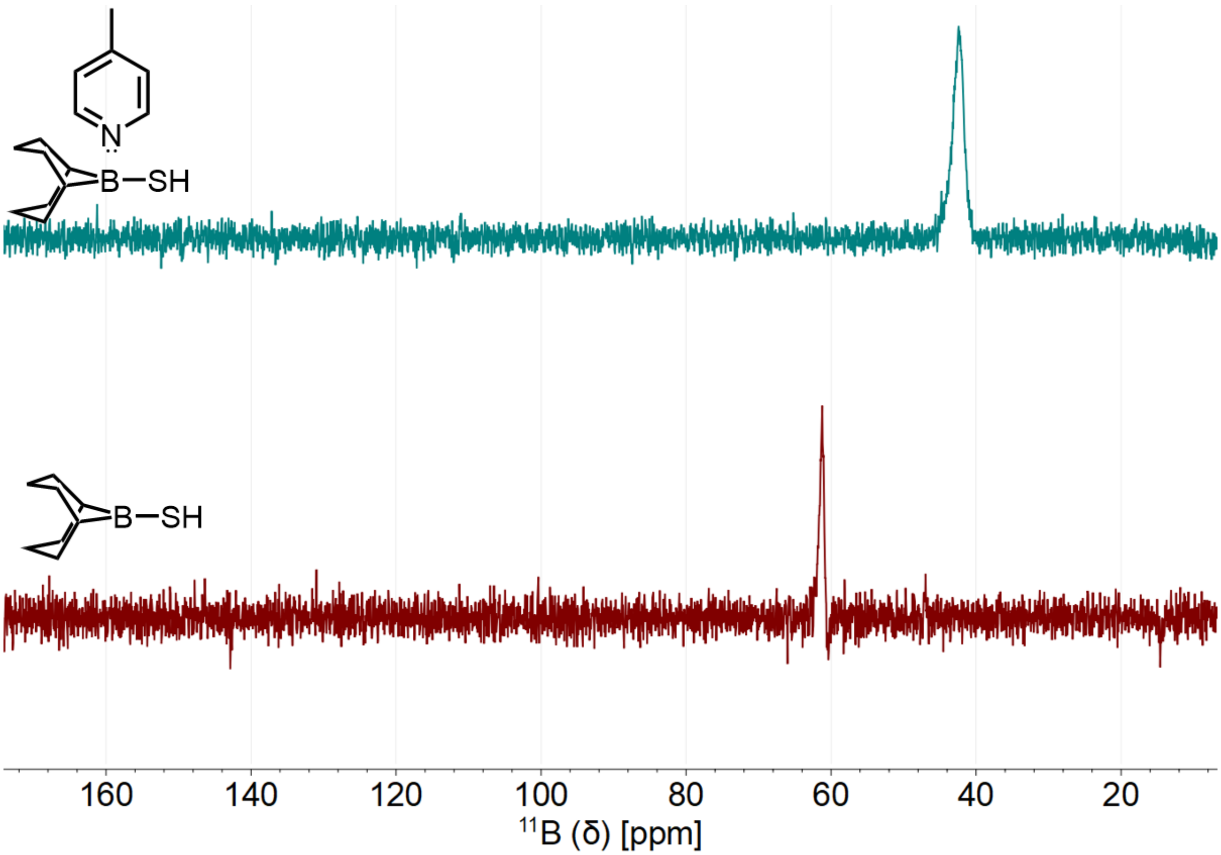

Figure S1. ^{11}B NMR spectrum of BBN-SH before (lower) and after (upper) adding picoline. Upon the addition of picolin, the electron density around the boron atom increases significantly. Therefore, the peak from the boron in BBN-SH at ~61 ppm shifts to up-field (~42 ppm).

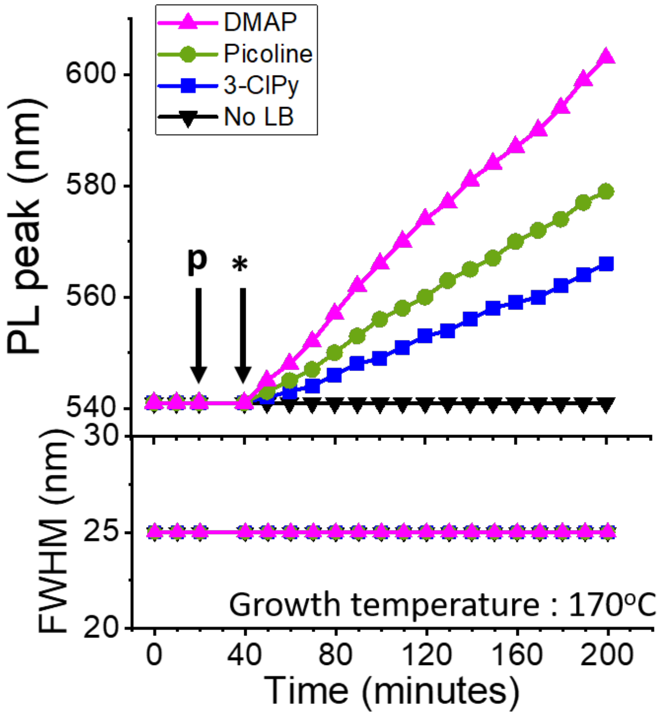

Figure S2. Modulated shell growth rates by varying LBs under constant temperature condition (170°C). No growth was observed when the precursors were injected ("p"). Injection of LBs initiates the shell growth (*). LBs yielding higher T_{act} show slower rate of shell growth (magenta triangle: DMAP, olive circle: picoline, blue square: 3-ClPy). The FWHM of PL spectra is maintained narrow during the shell growth, indicating that the tight size distribution of CdSe/CdS core/shell QDs is maintained. In the absence of LB, CdS shell deposition is not initiated (black flipped triangle).

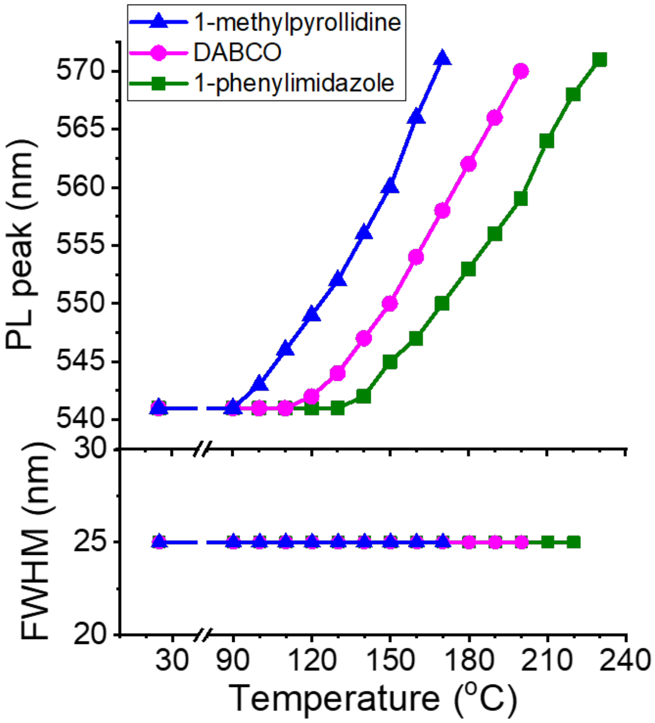

Figure S3. CdS shell growth using BBN-SH and non-pyridine based LBs. The reactivity of BBN-SH is modulated using LBs having different BF_3 affinities (1-methylpyrollidine(blue triangle), DABCO(magenta circle), 1-phenylimidazole(olive square)). The FWHM of PL spectra is maintained narrow during the shell growth, indicating that the tight size distribution of QDs is maintained.

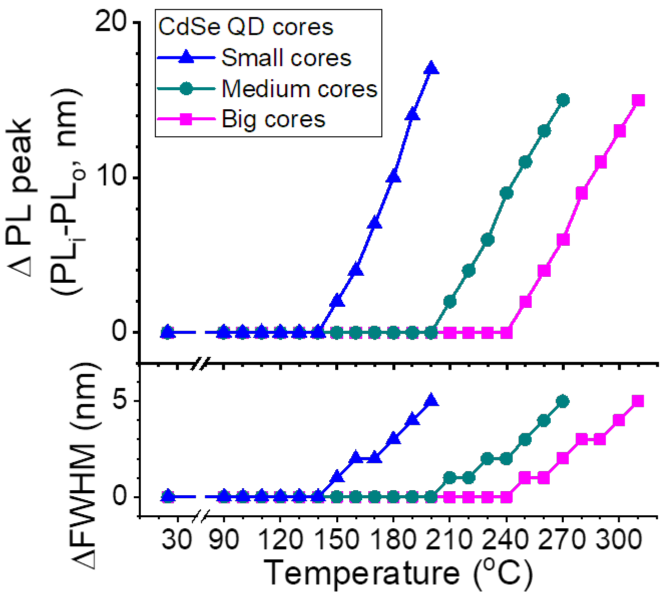

Figure S4. Melting temperature of CdSe QDs of different sizes. The peak and FWHM of PL spectra of the CdSe QD cores are monitored while increasing the temperature [small size (d: 1.8 nm, blue triangle), medium size (d: 3.6 nm, cyan circle), and big size (d: 4.6 nm, magenta square)]. A red-shifted PL peak and broadened FWHM indicate that CdSe QDs become unstable, leading to Ostwald ripening.

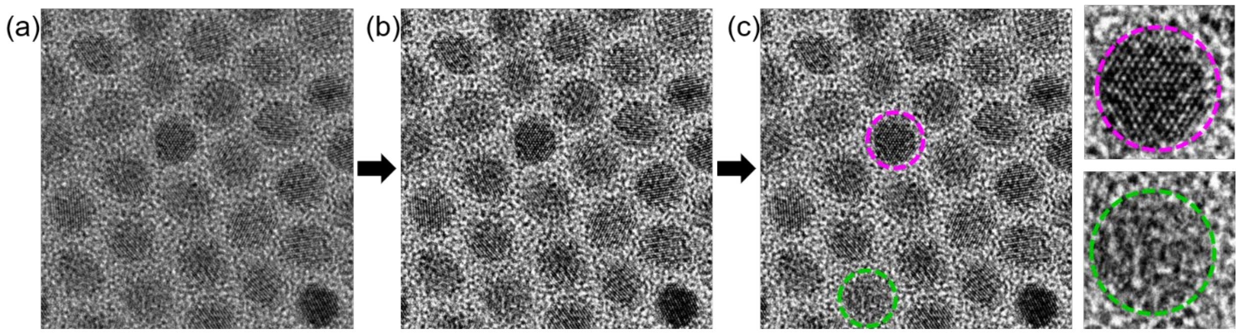

Figure S5. Flow of processing TEM images to count the number of QDs crystalline structures. After two steps of processing (a: TEM image without any correction, b: enhancing contrast 10%, c: smoothing), we count the number of QDs with a single crystal out of 300 nanoparticles, while excluding QDs with twin boundary

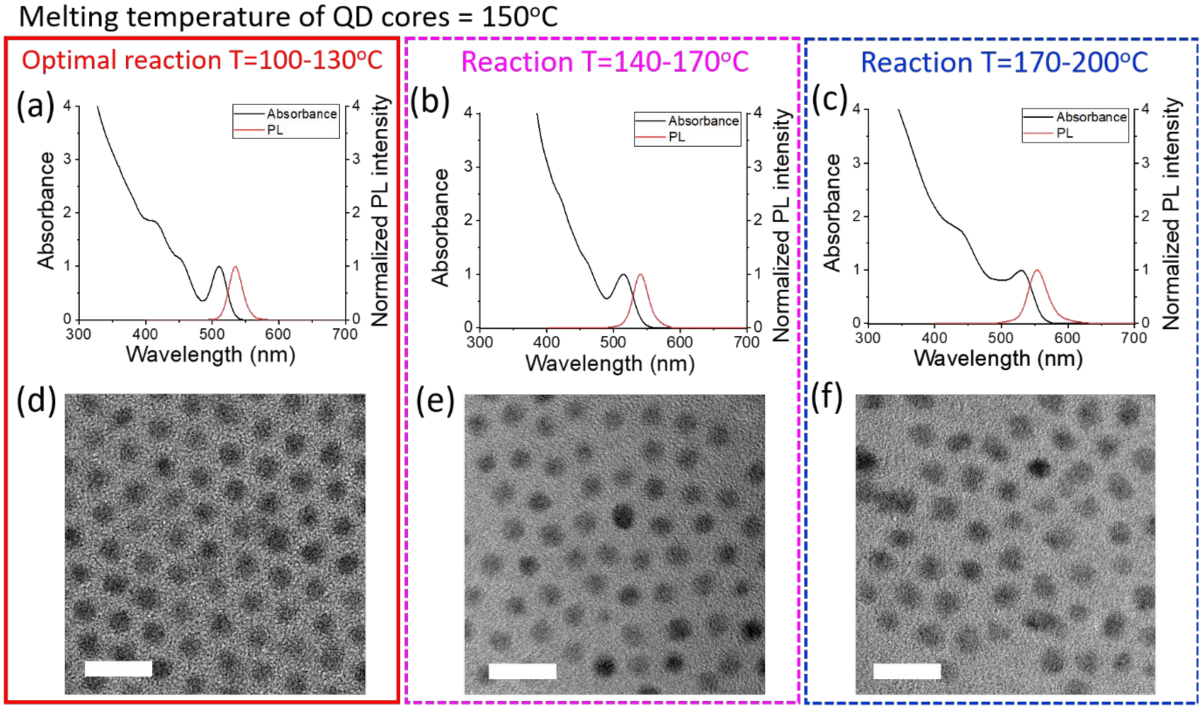

(g)

Reaction temperature	PL peak (nm)	FWHM (nm)	QY (%)	Crystallinity (%)
100-130ºC	536	25	75	92
140-170ºC	541	27	47	67
170-200ºC	554	33	60	71

Figure S6. Optimization of the reaction temperature by growing shells onto (sm)-CdSe QDs (T_m: 150ºC) using different LBs. The reaction temperature should be in the optimal range (10~20ºC lower than the melting temperature of QD cores). Shell growth at higher reaction temperatures results in lower QY, broad FWHM, undesired alloying, and irregular shapes. (a-c) The absorbance and PL spectra and (d-f) TEM images of (sm)-CdSe/CdS QDs (scale bar: 20 nm). CdS shell growth on (sm)-CdSe QD cores using [BBN-SH:DMAP] (optimal pair, a,d), [BBN-SH:picoline] (b,e), and [BBN-SH:3-ClPy] (c,f). (g) Summary of optical and structural quality of each QD sample.

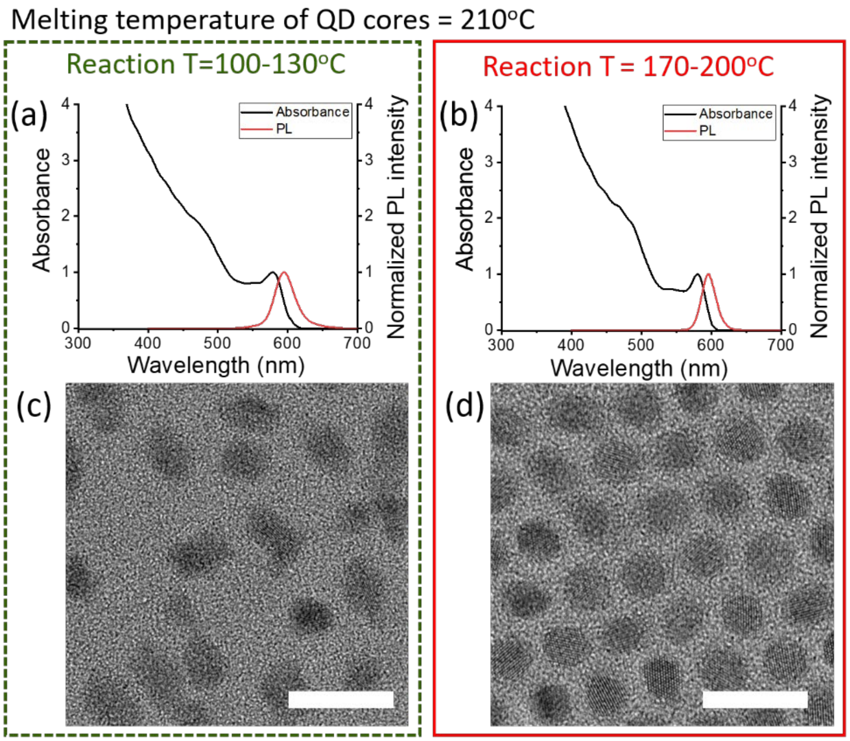

(e)

Reaction temperature	PL peak (nm)	FWHM (nm)	QY (%)	Crystallinity (%)
100-130ºC	595	32	52	42
170-200ºC	597	25	79	90

Figure S7. Optimization of the reaction temperature by growing shells onto medium CdSe QDs (T_m: 210ºC) using different LBs. The reaction temperature should be in the optimal range (10~20ºC lower than the melting temperature of QD cores). Shell growth at lower reaction temperatures result**s** in lower QY, broad FWHM, undesired alloying, and irregular shapes. (a,b) Absorbance and PL spectra and (c,d) TEM images of CdSe/CdS QDs after shell growth (scale bar: 20 nm). CdS shell growth on medium size CdSe QD cores using [BBN-SH:DMAP] (a,c) and [BBN-SH:3-ClPy] (optimal pair, b,d). Shell growth at the optimal temperature produce QDs having narrow FWHM, high QY, and regular shape and size. (e) Summary of the optical and structural quality of each QD sample.

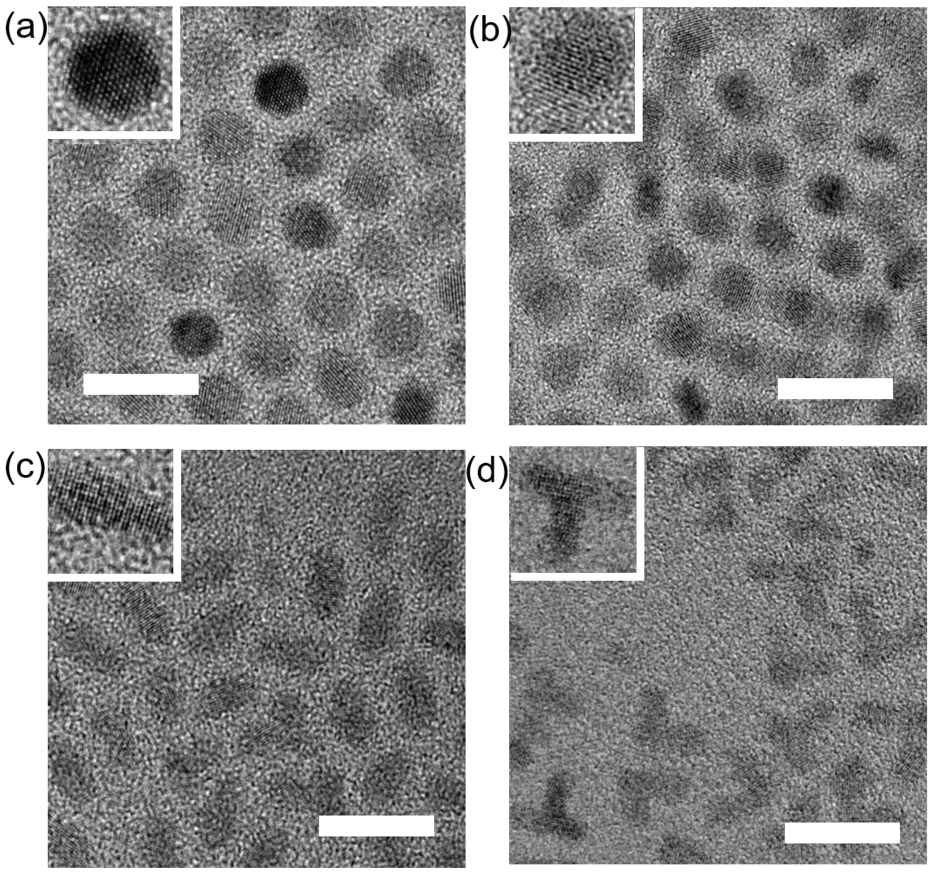

Figure S8. Both temperature and strongly coordinating ligands are required to achieve the asymmetric QD growth. TEM images (Inset: HRTEM images) of CdSe/CdS QDs synthesized using either wurtzite CdSe QD cores with either (a) [BBN-SH:4-CF_3Py] pair at 200ºC, (b) [BBN-SH:picoline] pair at 140ºC, or (c) [BBN-SH:picoline] pair at 140ºC in the presence of octadecylphosphonic acid. TEM images of CdSe/CdS QDs synthesized using zinc-blende CdSe QD cores with (d) [BBN-SH:picoline] pair at 140ºC in the presence of octadecylphosphonic acid. (Scale bar: 20 nm)

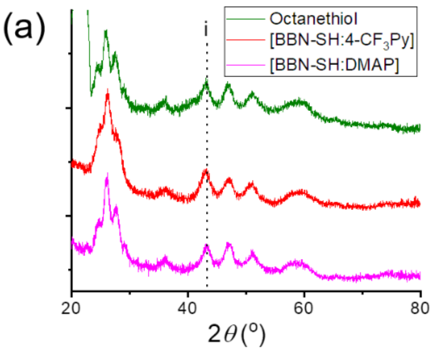

(b)

Precursor types	FWHM (degree)	FWHM (rad, x 10^{-2})	Peak position	Size of coherent volume (nm)
Octanethiol	1.64	2.87	43.08	5.20
[BBN-SH : 4-CF_3Py]	1.63	2.85	43.05	5.24
[BBN-SH : DMAP] (mismatched pair)	1.73	3.02	43.21	4.93

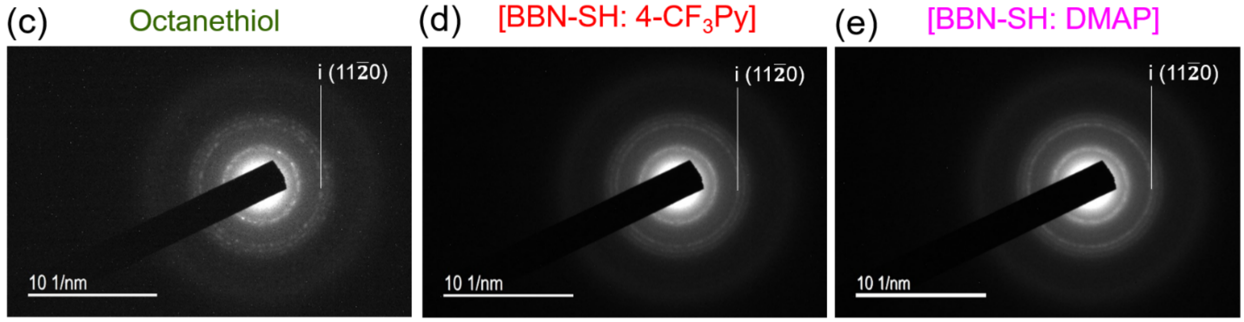

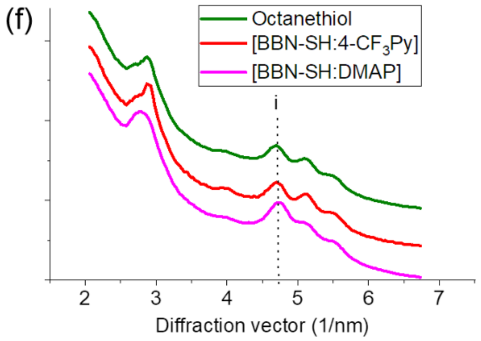

(g)

Precursor types	FWHM (1/nm)
Octanethiol	0.27
[BBN-SH : 4-CF_3Py]	0.28
[BBN-SH : DMAP] (mismatched pair)	0.39

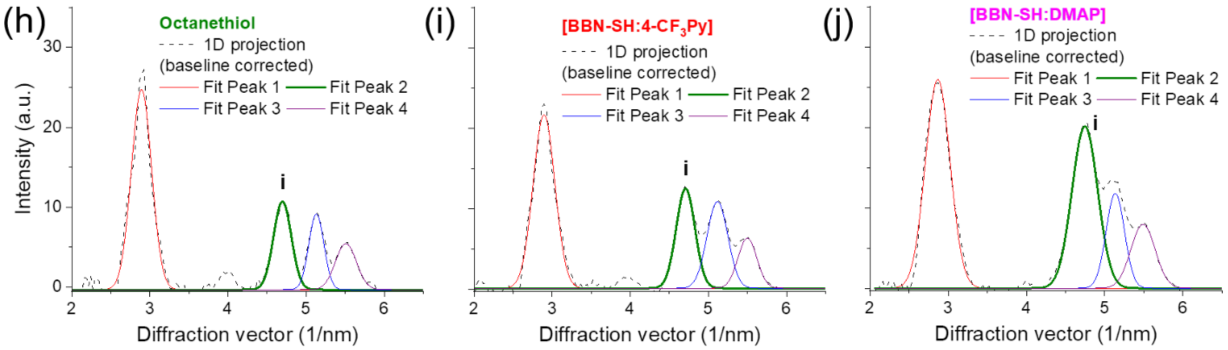

Figure S9. Higher precursor reactivity negatively impacts the crystallinity of QDs. (a) XRD data and (b) the summary of the CdS wurtzite(wz) (11$\bar{2}$0) peak ("i") and the size of coherent volume from (bg)-CdSe/CdS core/shell QDs synthesized by either octanethiol (olive), [BBN-SH:4-CF_3Py] pair (red), or [BBN-SH:DMAP] pair with 12 times slower injection (magenta). (c-e) Selected area electron diffraction (SAED) pattern (scale bar: 10 (1/nm)), (f) the projected 1D profile along the radius of diffraction ring pattern for resulted QD samples, (g) the FWHM of the arc from the CdS wurtzite(wz) (11$\bar{2}$0) peak ("i") from (bg)-CdSe/CdS core/shell QDs synthesized by either octanethiol (olive), [BBN-SH:4-CF_3Py] pair (red), or [BBN-SH:DMAP] pair with 12 times slower injection (magenta), and (h-j) the projected 1D profile after baseline correction and peaks with multiple Gaussian fitting.

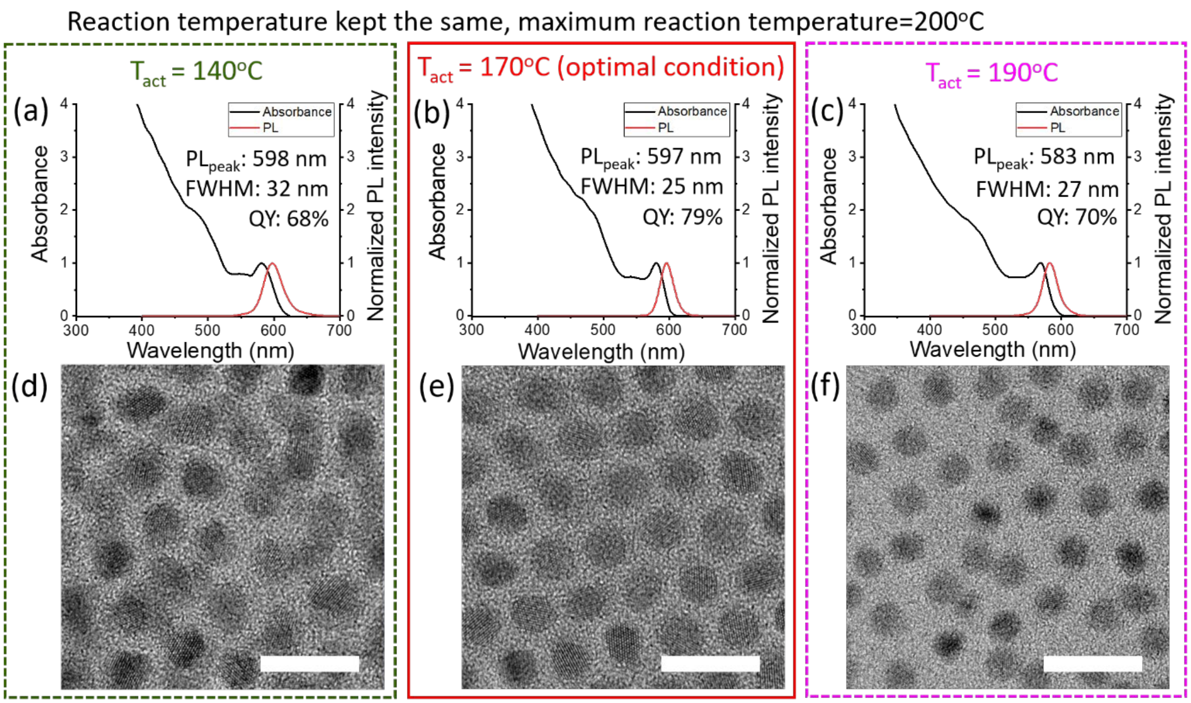

(g)

Activation temperature	PL peak (nm)	# of deposited shell	FWHM (nm)	QY (%)	Crystallinity (%)
140°C	598	7 MLs	32	68	72
170°C	597	7 MLs	25	79	90
190°C	583	5 MLs	27	70	53

Figure S10. Impact of precursor reactivity on the optical and structural quality of QDs. CdSe/CdS QDs was synthesized at the same reaction temperatures using BBN-SH and Lewis bases that have different activation temperature. Higher reactivity precursor results in irregular shape and broad FWHM, whereas lower reactivity precursor results the inefficient shell growth. (a-c) Absorbance and PL spectra and (d-f) the TEM images of CdSe/CdS QDs (scale bar: 20 nm). CdS shell growth onto the medium size CdSe QD cores at the same reaction temperature (200°C) using (a,d) [BBN-SH:picoline] (olive, T_{act}=140°C), (b,e) [BBN-SH:3-ClPy] (red, optimal condition, T_{act}=170°C), and (c,f) [BBN-SH:4-CyPy] (magenta, T_{act}=190°C). (g) Summary of the optical and structural quality of each QD sample.

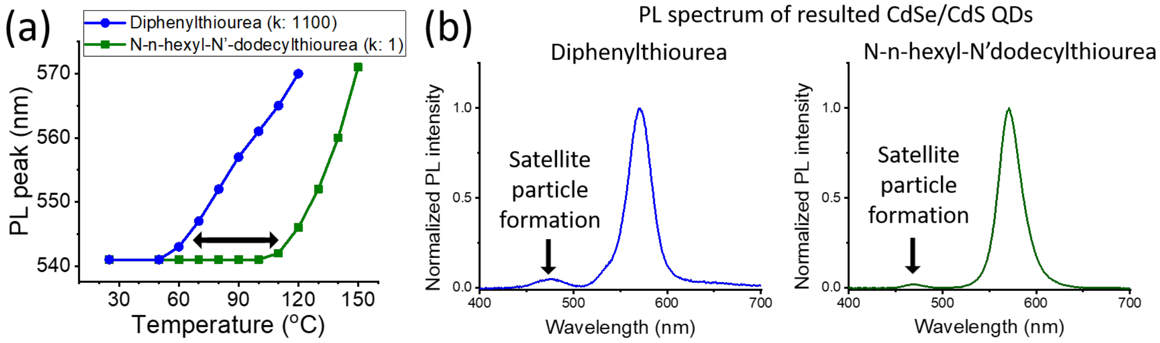

Figure S11. Characterization of the activation temperature of thiourea-based sulfur precursors. We examined two thioureas havinag either the highest (*k*: 1100) or the lowest conversion rate (*k*: 1). a) The activation temperature of thiourea precursors are low (60-110°C), making them undesired candidates for shell growth. b) The PL spectra of CdSe/CdS QDs (PL peak ~570 nm) produced with different thioureas indicates satellite particle formation.

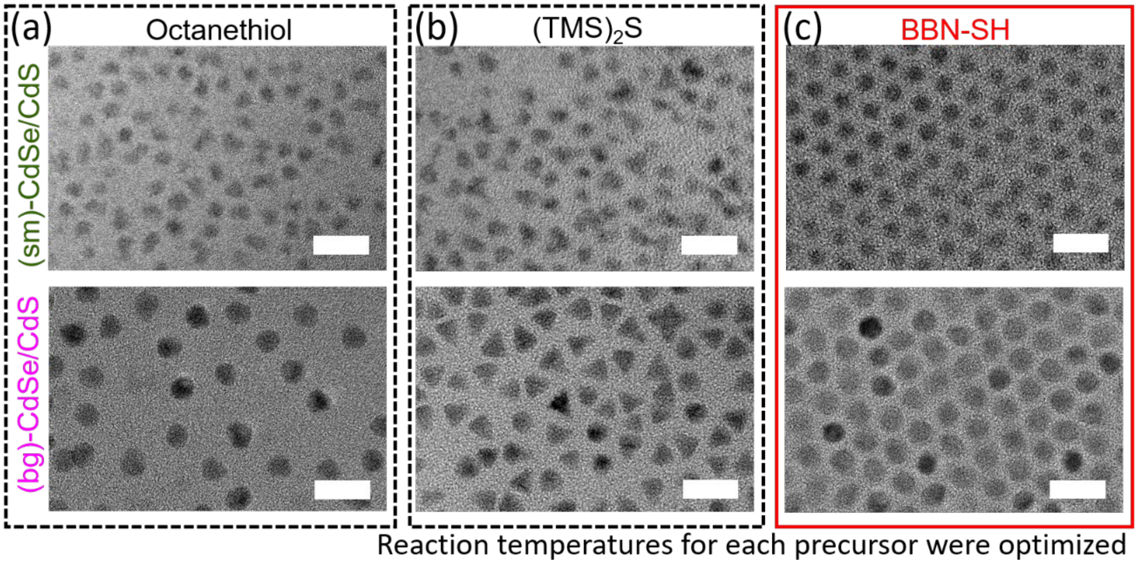

Figure S12. The wide field view of the TEM images in Figure 2. (a, b) The QDs produced with conventional precursors (a: octanethiol(OT), b: $(TMS)_2S$) show low crystallinity, poor uniformity in size, and irregular shape, except (bg)-CdSe/CdS QDs grown with OT. (c) [BBN-SH:LB] enables a systematic growth of high quality shells (scale bar: 20 nm).

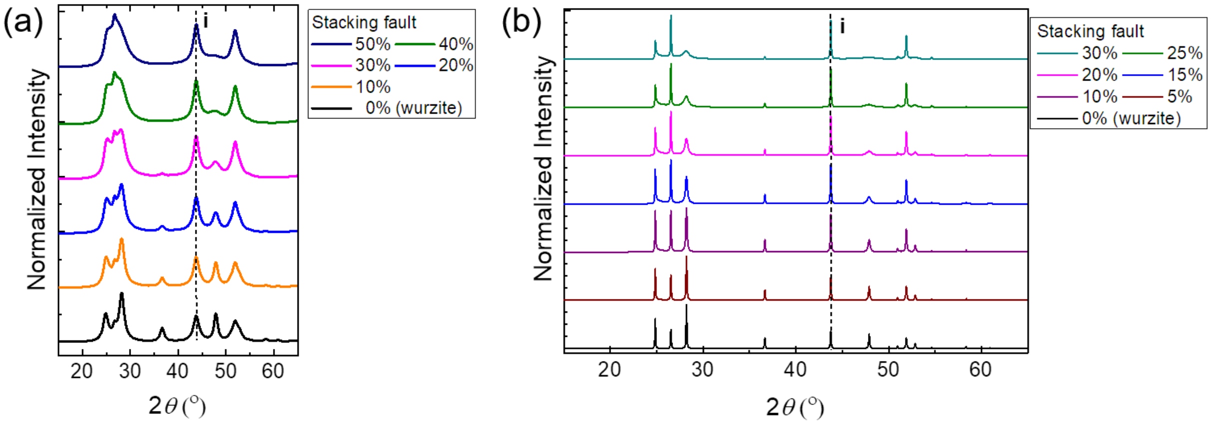

Figure S13. Stacking fault does not affect on the FWHM of the CdS wurtzite(wz) $(11\bar{2}0)$ peak. Simulated XRD data from (a) nanocrystals or (b) bulk comprised with CdS wurtzite crystal (host) and zinc-blende (defect). FWHM of $(11\bar{2}0)$ peak ("i") in simulated XRD data for both nanocrystal (1.4) and bulk (0.1) was maintained while varying the percentage of stacking faults.

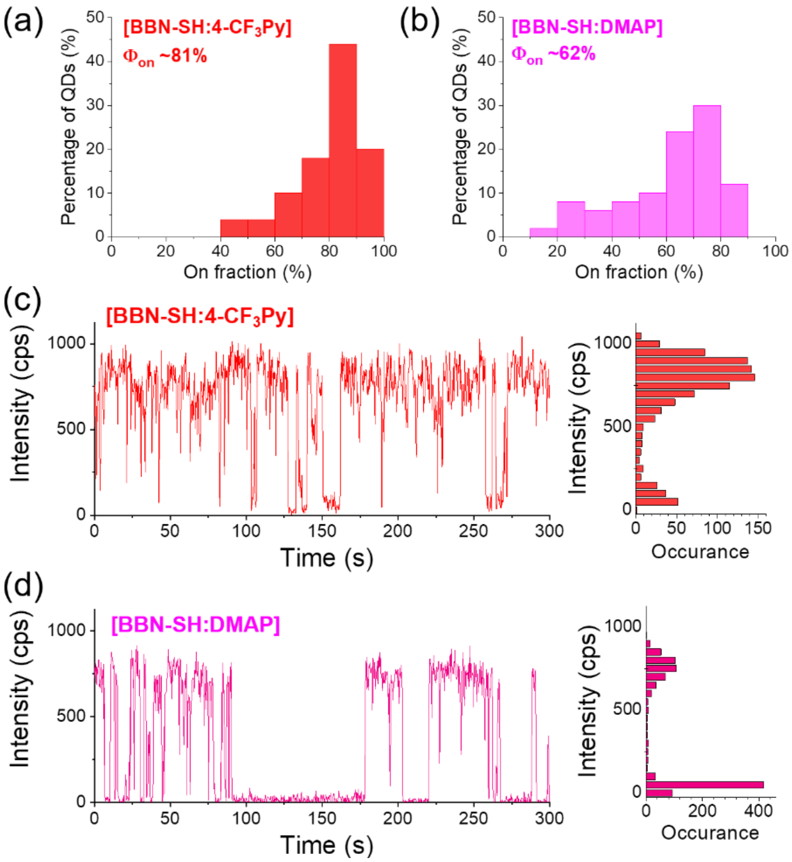

Figure S14. (a,b) Histogram of the blinking ON-time fraction for CdSe/CdS core/shell QDs synthesized from (a) optimal pair ([BBN-SH:4-CF_3Py]) and (b) unoptimal pair ([BBN-SH:DMAP]). (c,d) Representative single QD blinking traces and histogram of the PL intensity for CdSe/CdS core/shell QDs grown with either (c) optimal pair ([BBN-SH:4-CF_3Py]) and (d) unoptimal pair ([BBN-SH:DMAP]). The average ON-time fraction (Φ_{on}) for optimal pair (81%) is higher than one for unoptimal pair (62%).

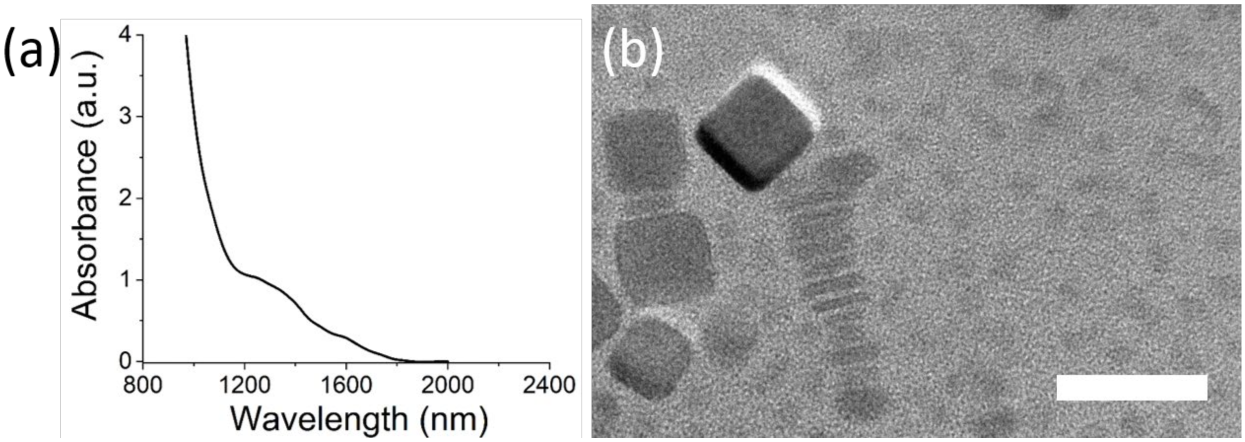

Figure S15. Synthesis of PbS QDs by the slow infusion of $(TMS)_2S$ as a sulfur precursor. (a) Absorbance spectrum and (b) TEM image of resulted PbS QDs (scale bar: 20 nm).

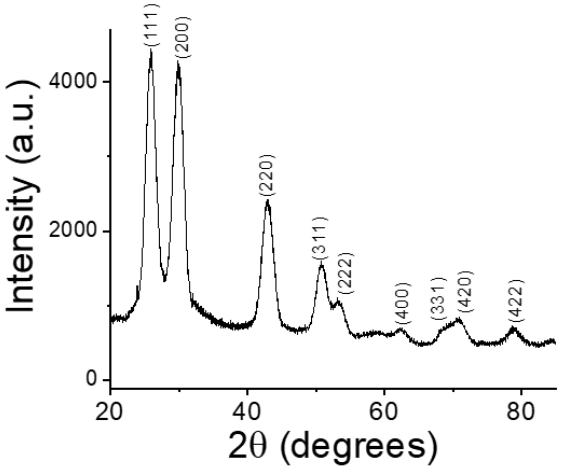

Figure S16. XRD data shows the highly crystallinity of PbS QDs prepared with BBN-SH and DMAP.

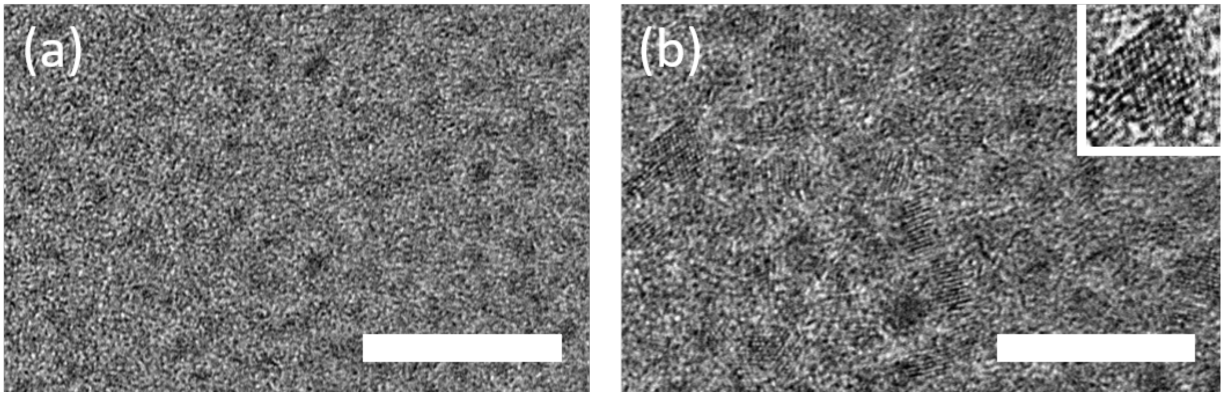

Figure S17. $CuInS_2$ QDs growth using BBN-SH:3-ClPy. The TEM images of (a) $CuInS_2$ neclei (d: 2.6 nm) and (b) $CuInS_2$ QDs grown with BBN-SH (d: 5.0 nm), scale bar: 20 nm.

Lewis base	Dimethylaminopyridine (DMAP) 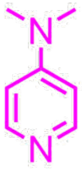	Picoline 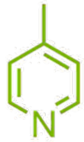	3-chloropyridine (3-ClPy) 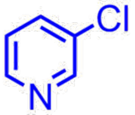	4-(trimethylfluoro) pyridine (4-CF_3Py) 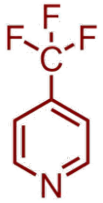	4-cyanopyridine (4-CyPy) 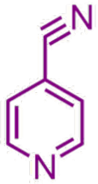
ΔH (kJ mol^{-1})	152	134	119	115	113

Table S1. BF_3 affinity scale for pyridine-based Lewis bases

Precursor type	Core size	Core PL peak (nm)	Core/shell PL peak (nm)	FWHM (nm)	QY (%)	QY w/ post treat (%)	Size (±std dev) (nm)	Circularity (±std dev)
BBN-SH	(sm)-CdSe	474	536	25	75	87	6.3 (±0.3)	0.80 (±0.08)
	(bg)-CdSe	590	627	24	82	91	9.1 (±0.4)	0.84 (±0.09)
$(TMS)_2S$	(sm)-CdSe	474	538	34	48	48	N/A	0.50 (±0.18)
	(bg)-CdSe	590	627	32	51	53	N/A	0.44 (±0.21)
Octanethiol	(sm)-CdSe	474	607	37	83	84	N/A	0.44 (±0.17)
	(bg)-CdSe	590	634	25	92	92	9.3 (±0.8)	0.82 (±0.12)

Table S2. Summary of the optical and physical properties of CdSe/CdS QDs grown with different precursors (BBN-SH, OT, and $(TMS)_2S$).

Precursor reactivity	PL peak (nm)	FWHM (nm)	QY (%)	Size distribution (%)	Circularity (±std dev)	Crystallinity (%)
Optimal	627	24	82	5	0.84 (±0.09)	92
High	627	33	51	21	0.62 (±0.15)	27
High (12 times slower injection)	627	27	65	15	0.78 (±0.12)	73

Table S3. Summary of the optical and the structural quality the QDs in Figure 3. High reactivity precursor results in QDs with low QY, broad FWHM and uncontrolled shape and size. Slower infusion (12 times) of the high reactivity precursor partially compensates the negative impact of suboptimal reactivity, however, the resulting QDs have significantly inferior structural quality compared to the QDs grown at the optimal condition.

CdSe QD core size	Reaction temperatures (°C)		
	BBN-SH	Octanethiol	$(TMS)_2S$
Small core	100-130	240-310	100-130
Medium core	170-200	240-310	100-130
Big core	200-230	240-310	100-130

Table S4. Temperature profile for shell deposition for the QDs presented in Figures 2.

Precursor type	Results from different characterization tools			
	Crystallinity (%), TEM	FWHM (degree) of $(11\bar{2}0)$ peak, XRD	FWHM (1/nm) of $(11\bar{2}0)$ peak, SAED pattern	Average ON-time fraction (%)
Octanethiol	92	1.64	0.27	N/A
[BBN-SH : 4-CF_3Py]	92	1.63	0.28	81
[BBN-SH : DMAP] (mismatched pair)	73	1.73	0.39	62

Table S5. Summary of crystallinity and single QD blinking measurements of each QD sample. From TEM analysis in Figure S5, the percentage of crystalline structure through the entire QD were obtained. XRD and SAED patter analyses show similar crystallinity for QDs from [BBN-SH:4-CF3Py] (optimal pair) with octanethiol case. Larger FWHM from both XRD and SAED pattern and smaller average ON-time fraction confirm the inferior crystallinity of QDs prepared by unoptimal pair ([BBN-SH:DMAP]).

	Element		
	Cu	In	S
Experimental value (at%)	24.8	24.5	50.7
Theoretical value (at%)	25.0	25.0	50.0

Table S6. Atomic ratio of Cu:In:S found in the resulted $CuInS_2$ QDs determined by ICP-OES

<References>

1 Hendricks, M. P., Campos, M. P., Cleveland, G. T., Plante, I. J. L. & Owen, J. S. A Tunable library of substituted thiourea precursors to metal sulfide nanocrystals. *Science* **348**, 1226-1230 (2015).

2 Hamachi, L. S. et al. Precursor reaction kinetics control compositional grading and size of $CdSe_{1-x}S_x$ nanocrystal heterostructures. *Chem. Sci.* **10**, 6539-6552 (2019).

3 Köster, R., Seidel, G., Siebert, W. & Gangnus, B. in *Inorg. Synth.* Vol. 29 (Wiley, New Jersey, 1992).

4 Han, H.-S. et al. Development of a bioorthogonal and highly efficient conjugation method for quantum dots using tetrazine−norbornene cycloaddition. *J. Am. Chem. Soc.* **132**, 7838-7839 (2010).

5 Chen, O. et al. Synthesis of metal–selenide nanocrystals using selenium dioxide as the selenium precursor. *Angew. Chem. Int. Ed.* **47**, 8638-8641 (2008).

6 Moreels, I. et al. Size-dependent optical properties of colloidal PbS quantum dots. *ACS Nano* **3**, 3023-3030 (2009).

7 Jara, D. H., Yoon, S. J., Stamplecoskie, K. G. & Kamat, P. V. Size-dependent photovoltaic performance of $CuInS_2$ quantum dot-sensitized solar cells. *Chem. Mater.* **26**, 7221-7228 (2014).

8 Manna, L., Wang, Cingolani, R. & Alivisatos, A. P. First-principles modeling of unpassivated and surfactant-passivated bulk facets of wurtzite CdSe: a model system for studying the anisotropic growth of CdSe nanocrystals. *J. Phys. Chem. B* **109**, 6183-6192 (2005).

9 Rempel, J. Y., Trout, B. L., Bawendi, M. G. & Jensen, K. F. Density functional theory study of ligand binding on CdSe (0001), ($000\bar{1}$), and ($11\bar{2}0$) single crystal relaxed and reconstructed surfaces: implications for nanocrystalline growth. *J. Phys. Chem. B* **110**, 18007-18016 (2006).
